# Pulmonary Involvement and Leptospirosis, Greece

**DOI:** 10.3201/eid1505.080270

**Published:** 2009-05

**Authors:** Anna Papa, Dionysia Theoharidou, Antonis Antoniadis

**Affiliations:** Aristotle University of Thessaloniki, Thessaloniki, Greece

**Keywords:** Zoonoses, bacteria, leptospirosis, pulmonary, hemorrhage, Greece, letter

**To the Editor:** Since the leptospirosis outbreak associated with pulmonary hemorrhage in Nicaragua in 1995 (*1*), pulmonary manifestations of leptospirosis are often recognized in many countries; reported incidence has ranged from 20% to 70% (*2**–**4*). The severe pulmonary form of leptospirosis is accompanied by pulmonary hemorrhage, which directly results in high death rates (*2**,**5*). In Greece (population 11 million), leptospirosis cases in humans occur every year, usually from June to November (summer and autumn), with a peak in August. The annual incidence rate of the disease is 3 cases per 1 million population (*6*). Clinical presentation varies from a flu-like syndrome to Weil disease, which includes jaundice, renal failure, and hemorrhagic complications. Studies on leptospirosis in Greece have been limited, and no reports have focused on pulmonary involvement.

During 1998–2007, we tested samples from 650 patients with suspected leptospirosis or hemorrhagic fever with renal syndrome (i.e., hantavirus infection). Various hospitals of northern Greece sent these samples to our laboratory (a World Health Organization Collaborating Center for Reference and Research on Arboviruses and Hemorrhagic Fever) for analysis. Because both diseases are endemic to Greece and have similar clinical, epidemiologic, and seasonal characteristics (*7*), all samples sent to our laboratory for testing either for leptospirosis or for hantavirus infection are always tested for both (*8*).

Leptospirosis was confirmed for 123 patients, 10 (8.1%) of whom died (Table). For 72 case-patients, paired samples were available. A commercial ELISA (Leptospira IgG/IgM, Institute Virion/Serion GmbH, Würzburg, Germany) was used to detect immunoglobulin (Ig) G and IgM against *Leptospira* spp.. A nested PCR, which amplifies a 289-bp fragment of the 16S rDNA gene, was used to detect bacterial DNA (*9*). IgM concentrations >20 U/mL indicated acute infection. Samples with borderline results were tested in parallel with a second sample taken from the patient 1 week later. IgG concentrations were considered only for paired samples, and a case was considered as acute leptospirosis when a >4-fold titer rise of IgG, or IgG seroconversion, was detected. When samples were taken before the sixth day of illness, initial diagnosis was achieved by PCR. In 6 of 10 fatal cases, leptospirosis was diagnosed only by PCR because antibodies were not detectable. Epidemiologic and clinical information for patients was collected from chart review, following a protocol approved by the medical school review board.

**Table:** Leptospirosis cases and pulmonary involvement, Greece, 1998–2007

Year	No. cases (no. fatal cases)	No. cases with pulmonary involvement (no. fatal cases)
1998	12 (0)	0 (0)
1999	9 (2)	3 (0)
2000	7 (1)	1 (0)
2001	11 (0)	1 (0)
2002	13 (2)	4 (2)
2003	13 (1)	6 (1)
2004	20 (3)	10 (3)
2005	14 (1)	3 (1)
2006	17 (0)	5 (0)
2007	7 (0)	2 (0)
Total	123 (10)	35 (7)

All 123 patients resided in northern Greece. Most (82.1%) were male; patients were 6–83 years of age (median 51 years). Fifty-two patients were farmers; 9 were sewer workers; and 6 and 2 patients, respectively, reported gardening and other recreational exposures before becoming ill. Cases occurred in all months; 27% occurred in August. Fever, as well as elevated levels of serum urea and creatinine, occurred in all patients; 55 (44.7%) had jaundice, and 46 (37.4%) had thrombocytopenia. Weil disease was present in 27 (22%). In our case-series, jaundice appeared to be a common sign, in contrast to recent studies in other countries in which the icteric form of the disease was observed in only 10% of cases (*10*). Median age of the 10 patients who died was 50 years (range 39–78 years). Half of the patients who died had the icteric form of the disease, 4 had the typical signs and symptoms of Weil disease, and 1 had central nervous system involvement.

Thirty-five (28.5%) patients had pulmonary signs and symptoms, either when admitted to the hospital or during hospitalization. Eight of these had acute respiratory distress syndrome, 6 had multiple organ dysfunction syndrome, and 6 had acute respiratory insufficiency; the remainder had hemoptysis and/or dyspnea, according clinician notes in the medical charts. More than half of the patients had abnormal radiographic findings, mainly bilateral bronchoalveolar infiltrations. Seven (20%) of the 35 case-patients with pulmonary involvement died, a significantly higher death rate than that for case-patients without pulmonary involvement (3.4%, p<0.01). Respiratory symptoms were recognized during the first phase of the disease, as other studies have reported (*10*). We found no significant difference in death rates between males and females (p = 0.629).

Pulmonary involvement seems to be common and associated with a high death rate for patients with severe leptospirosis cases in our setting. Clinicians in Greece should include leptospirosis in the differential diagnosis of syndromes with associated pulmonary manifestations.
